# Association study of the complement component *C4* gene and suicide risk in schizophrenia

**DOI:** 10.1038/s41537-024-00440-w

**Published:** 2024-02-10

**Authors:** Mahbod Ebrahimi, Kowsar Teymouri, Cheng C. Chen, Ayeshah G. Mohiuddin, Jennie G. Pouget, Vanessa F. Goncalves, Arun K. Tiwari, Clement C. Zai, James L. Kennedy

**Affiliations:** 1 Tanenbaum Centre for Pharmacogenetics, Molecular Brain Science, Campbell Family Mental Health Research Institute, Centre for Addiction and Mental Health, Toronto, Canada; 2 Institute of Medical Science, University of Toronto, Toronto, Canada; 3 Department of Psychiatry, University of Toronto, Toronto, Canada; 4 Department of Pharmacology and Toxicology, University of Toronto, Toronto, Canada; 5 Laboratory Medicine and Pathobiology, University of Toronto, Toronto, Canada

**Keywords:** Molecular neuroscience, Genetics of the nervous system

## Abstract

Schizophrenia is a severe mental illness and a major risk factor for suicide, with approximately 50% of schizophrenia patients attempting and 10% dying from suicide. Although genetic components play a significant role in schizophrenia risk, the underlying genetic risk factors for suicide are poorly understood. The complement component *C4* gene, an immune gene involved in the innate immune system and located in the major histocompatibility complex (MHC) region, has been identified to be strongly associated with schizophrenia risk. In addition, recent findings have also suggested that the MHC region has been associated with suicide risk across disorders, making *C4* a potential candidate of interest for studying suicidality in schizophrenia patients. Despite growing interest in investigating the association between the *C4* gene and schizophrenia, to our knowledge, no work has been done to examine the potential of *C4* variants as suicide risk factors in patients with schizophrenia. In this study, we investigated the association between different *C4* copy number variants and predicted *C4* brain expression with suicidal outcomes (suicide attempts/suicidal ideation). We directly genotyped 434 schizophrenia patients to determine their *C4*A and *C4*B copy number variants. We found the *C4*AS copy number to be marginally and negatively associated with suicide risk, potentially being protective against suicide attempts (OR = 0.49; *p* = 0.05) and suicidal ideation (OR = 0.65; *p* = 0.07). Furthermore, sex-stratified analyses revealed that there are no significant differences between males and females. Our preliminary findings encourage additional studies of *C4* and potential immune dysregulation in suicide.

## Introduction

Schizophrenia is a severe mental illness and a major risk factor for suicide, especially in the early stages of the disease. Suicide is among the largest causes of death among schizophrenia patients, with approximately 50% attempting and 10% dying from suicide^1,2^. Although suicide is a consequence of complex psycho-social factors, the predisposition to suicidality is at least partly explained by genetics. Heritability estimates captured by twin studies range from 30 to 55% for suicidal thoughts and behaviours^3^. In the context of psychiatric disorders, shared heritability between schizophrenia and suicide attempt/ideation has been demonstrated via polygenic risk scores and genetic correlation analyses, revealing a strong positive association^4,5^. Two recent large-scale genome-wide association studies (GWAS) in the International Suicide Genetics Consortium (ISGC) and Million Veteran Program (MVP) have demonstrated promising and replicable results. In a psychiatric cohort, the ISGC (total *N* = 549,743; 29,782 cases) detected two genome-wide significant loci associated with suicide attempts on the major histocompatibility complex (chromosome 6, index SNP rs71557378, *p* = 1.97 × 10^−^^8^) and an intergenic locus on chromosome 7 (index SNP rs62474683, *p* = 1.91 × 10^−^^10^), with the latter locus independently replicated by the MVP study^6,7^. Leveraging from these two studies, the largest and most recent meta-analysis to date (total *N* = 958,896; 43,871 suicide attempt/death cases) has identified 12 genome-wide significant loci, notably a locus in the major histocompatibility complex (MHC)^8^.

In addition to the new potential association with suicide risk, the MHC region has been long implicated in schizophrenia GWAS studies^9–12^. The most recent and largest schizophrenia GWAS study (*N* = 74,776 cases and 101,023 controls) has identified 287 genetic regions associated with schizophrenia, with the MHC region, by far showing the strongest association^12^. The MHC region, spanning four million base pairs on chromosome 6, is best known for its role in immunity, with genes encoding for human leukocyte antigens (HLA) and many others participating in immune functioning (e.g., complement genes)^13^. Having hundreds of genes with complex linkage disequilibrium and high variability in the MHC region has made it particularly challenging to find a functional allele to explain the schizophrenia-MHC association. However, this association has been partly explained by a landmark study on complex variations of the complement component 4 (*C4*) gene, which codes for an activator of the complement system^14^.

**Fig. 1:**
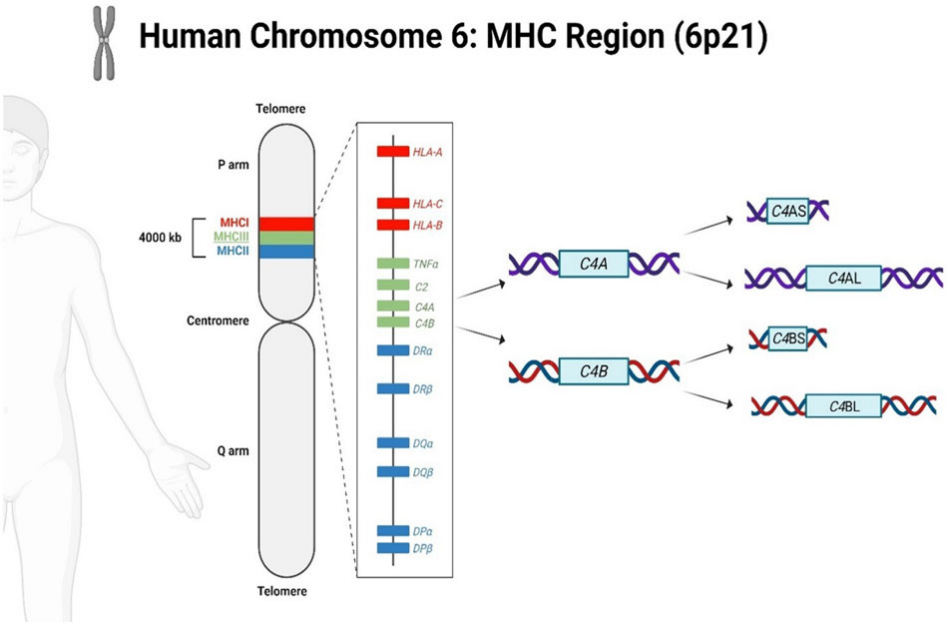
Schematic of the MHC region on chromosome 6, and the *C4* gene compound structural forms (created by Biorender.com).

The human *C4* gene, located in the MHC class III region (chr 6: 31.98–32.04 Mb), has a sophisticated genetic architecture and is present as two functionally distinct isotypes, *C4*A and *C4*B^15,16^. Each functional *C4* isotype (*C4*A, *C4*B) could be further segregated into long (*C4*L) or short (*C4*S) genomic forms^17^ depending on the presence or absence of a human endogenous retroviral type K (HERV-K) insertion in intron 9 of the *C4* gene^18^, resulting in four possible combinations (*C4*AL, *C4*BL, *C4*AS, *C4*BS) (Fig. 1). In addition, different individuals could have varying copy numbers of *C4*A and *C4*B genes, giving rise to at least 22 different haplotypes^15^. By determining the copy number of *C4* structural haplotypes, Sekar et al. found that the four common haplotypes (AL-AL, AL-BS, AL-AL, and BS) are associated with varying degrees of schizophrenia risk^16,19^. Specifically, in the context of *C4*, higher brain *C4*A expression is a predictor of increased schizophrenia risk, with the AL-AL haplotype conferring the highest risk (OR = 1.3)^14^.

In support of Sekar et al.’s findings, other studies have also shown that higher *C4*A expression is observed in schizophrenia post-mortem brains compared to healthy controls^20–22^. This higher *C4*A expression has been associated with more severe psychopathology symptoms, likely due to excessive complement activity in the brain of schizophrenia patients which disrupts the normal synaptic pruning process^20,23^. In line with these results, *C4*B and *C4*S deficiency has also been reported in schizophrenia patients^21,24,25^, and there is an inverse relationship between *C4*A and *C4*B copy number variations; similarly, with *C4*L and *C4*S^19^. However, it should be noted that there have been mixed results with regard to *C4*B deficiency across different ancestries. These findings warrant the question of whether *C4*B/*C4*S might have a protective effect against schizophrenia risk, whereas *C4*A/*C4*L might lead to deleterious effects.

Overall, fine-mapping studies suggest that the *C4* gene is a critical driver of the association between the MHC region and schizophrenia. Given the recent association of the MHC region with suicide, we sought to explore the potential role of different *C4* variants in suicide risk in our sample of schizophrenia patients. In this study, we explored the relationship between *C4* genetic variants and predicted *C4* brain expression with suicide risk (suicide attempt/ideation) in our Toronto schizophrenia cohort. Moreover, we examined the sex-specific effects of *C4* variants on suicidality.

## Results

**Fig. 2:**
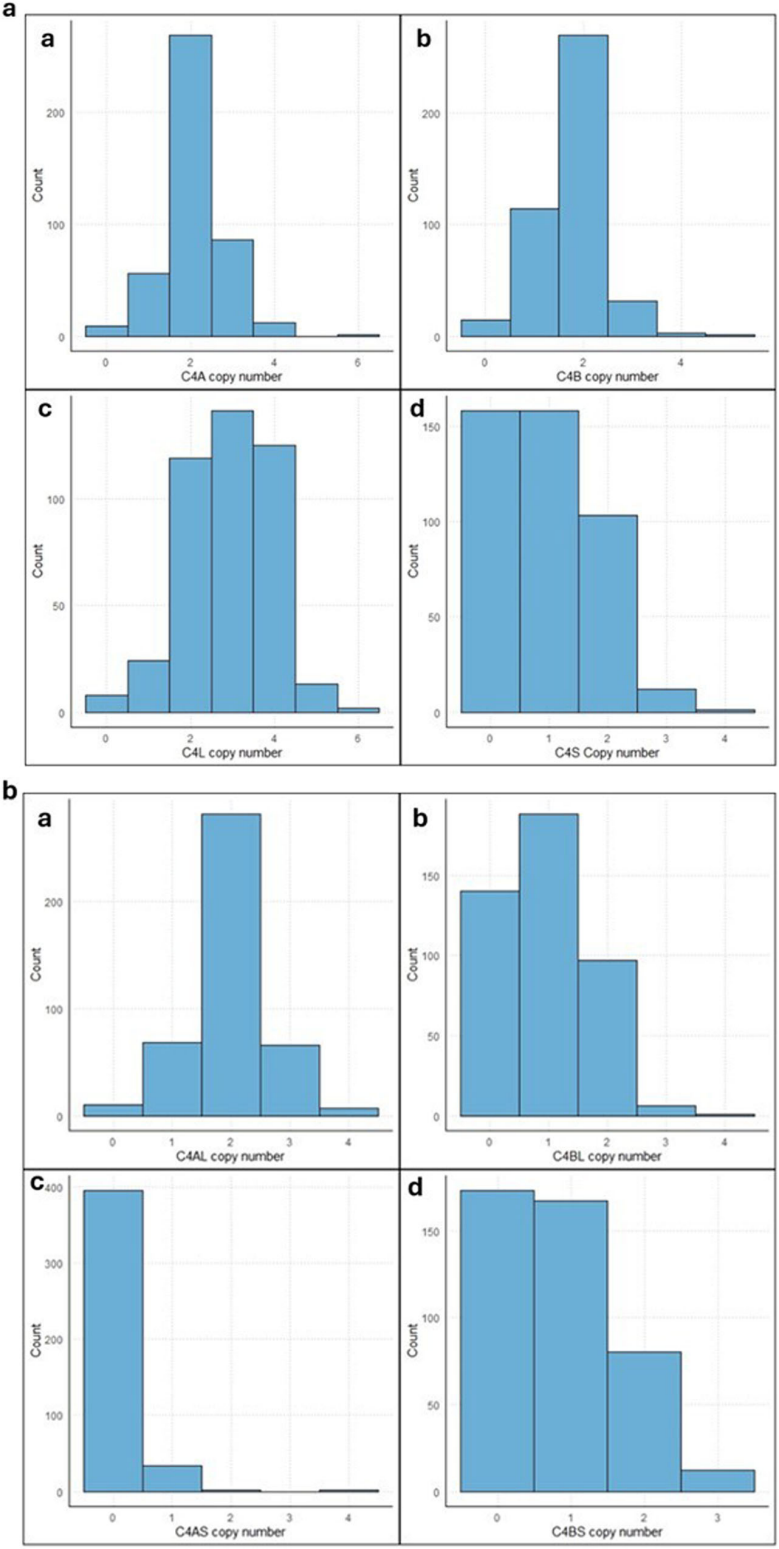
Distribution of *C4* structural and compound structural forms in our sample (*N* = 432). **a** Distribution of *C4* structural forms a) *C4*A, b) *C4*B, c) *C4*L, d) *C4*S (*N* = 432)**. b** Distribution of *C4* compound structural forms a) *C4*AL, b) *C4*BL, c) *C4*AS, d) *C4*BS (*N* = 432).

**Fig. 3:**
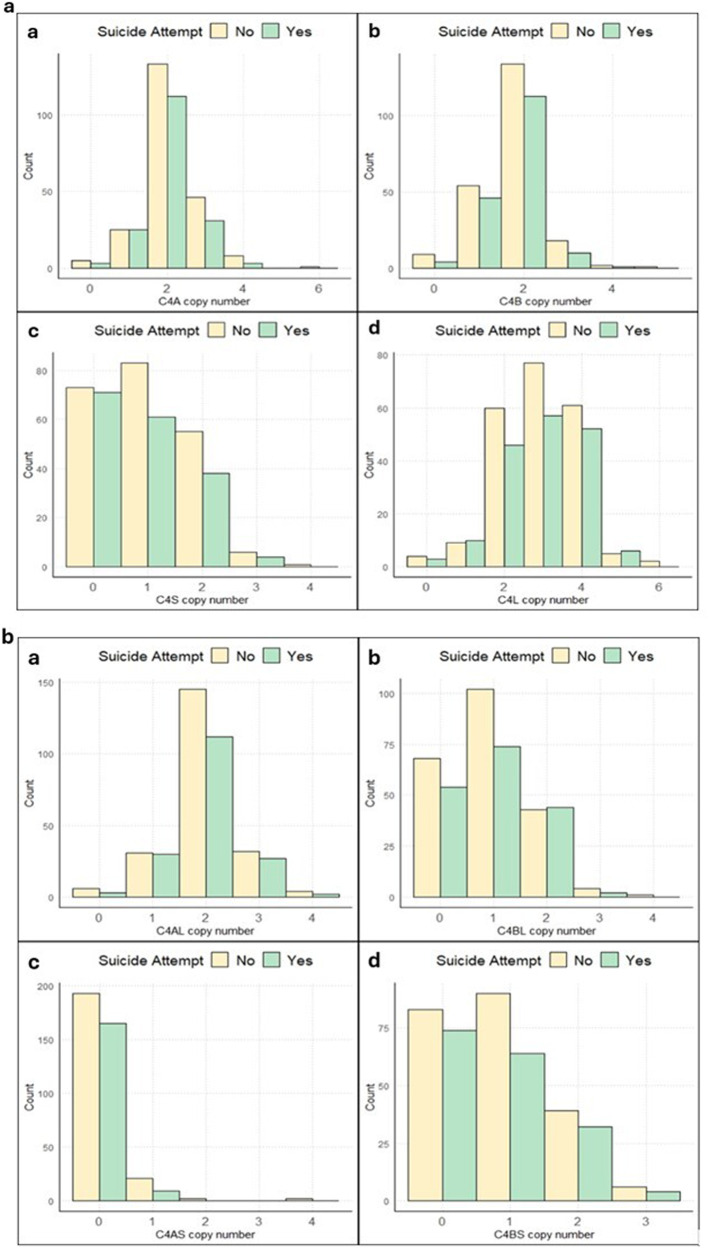
Distribution of *C4* structural and compound structural forms categorized by suicide attempt. **a** Distribution of *C4* structural forms a) *C4*A, b) *C4*B, c) *C4*L, d) *C4*S categorized by suicide attempt (*N* = 391). **b** Distribution of *C4* compound structural forms a) *C4*AL, b) *C4*BL, c) *C4*AS, d) *C4*BS categorized by suicide attempt (*N* = 391).

The distribution of *C4* structural forms (*C4*A, *C4*B, *C4*L, *C4*S) and compound structural forms (*C4*AL, *C4*AS, *C4*BL, *C4*BS) could be found in Fig. 2a, b. Moreover, the distribution of *C4* structural forms categorized by suicide attempt can be found in Fig. 3a, b. From the total *N* = 434 subjects with *C4* genotype data, only subjects with complete phenotypic data were included in the analysis.

### Association between *C4* and suicide attempt and suicidal ideation

**Fig. 4:**
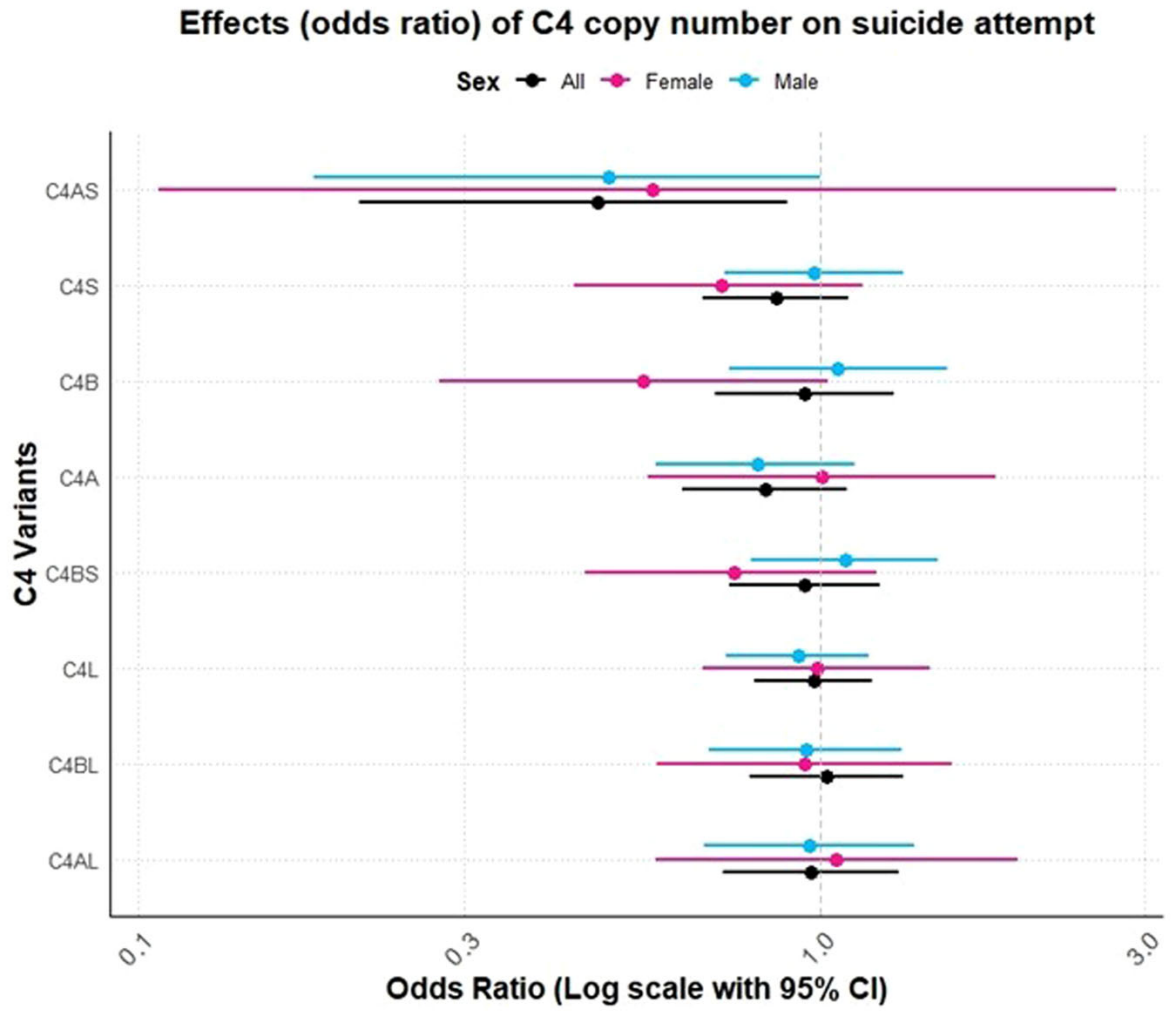
Effects (odds ratio) of *C4* copy number on suicide attempt. The odds ratios and confidence interval (CI) based on our logistic regression model for the entire sample (*N* = 391), then males (in blue, *N* = 283) and females (in pink, *N* = 108) separately.

Suicide attempts and suicidal ideation were recorded in 391 and 394 subjects, respectively. Figure 4 shows the odds ratios (OR) and their precision based on our logistic regression model for the entire sample, then males and females separately, while Tables 1, 2.1, and 2.2 demonstrate the same results in tabular form, with unadjusted (Supplementary Table 2) and adjusted regression p-values (p-values were not corrected for multiple tests). We see that, in general, there is not much evidence of an association between increasing *C4* copy number and suicide attempt and suicidal ideation, except possibly for *C4*AS, where we see some evidence of a negative association for suicide attempts (OR for total sample =0.491, Beta = −0.710, 95% CI: [0.217–0.936], *p* = 0.056, Table 1) and suicidal ideation (OR for total sample = 0.651, Beta = 0.814, 95% CI: [0.355–1.099], *p* = 0.074, Table 1), characterized by a drop in the prevalence of suicide attempts and suicidal ideation as the copy number of *C4*AS increases. We also notice that Fig. 4 seems to show a general tendency for negative associations across most of the variants.

**Table 1:** Logistic regression analyses between *C4* variants and suicide attempt/ideation in males and females with age, sex, and ancestry as covariates

Males and Females (*N* = 391)								
C4 Variant	Suicide Attempt				Suicidal Ideation			
	*P*-value	Odds Ratio	95% CI	Beta	*P*-Value	Odds Ratio	95% CI	Beta
C4A	0.360	0.876	[0.658–1.160]	−0.131	0.465	0.900	[0.677–1.191]	−0.104
C4B	0.453	0.889	[0.652–1.208]	−0.117	0.360	0.865	[0.632–1.177]	−0.144
C4L	0.873	0.983	[0.805–1.201]	−0.016	0.311	0.900	[0.734–1.100]	−0.104
C4S	0.221	0.855	[0.664–1.097]	−0.156	0.895	0.983	[0.766–1.263]	−0.016
C4AL	0.904	1.018	[0.752–1.379]	0.018	0.999	0.999	[0.735–1.357]	−0.0001
C4AS	0.056	0.491	[0.217–0.936]	−0.710	0.074	0.651	[0.355–1.099]	0.814
C4BL	0.952	0.991	[0.759–1.292]	−0.008	0.367	0.884	[0.675–1.154]	−0.122
C4BS	0.609	0.934	[0.720–1.210]	−0.067	0.740	1.044	[0.807–1.356]	0.043
C4A Expression	0.360	0.776	[0.448–1.332]	−0.253	0.248	0.724	[0.415–1.245]	−0.322
C4B Expression	0.528	0.902	[0.652–1.242]	−0.103	0.455	0.885	[0.641–1.215]	−0.121

**Table 2:** 1. Sex-stratified logistic regression analysis between *C4* variants and suicide attempt/ideation in males with age and sex as covariates

1								
Males (*N* = 283)								
C4 Variant	Suicide attempt				Suicidal Ideation			
	*P*-Value	Odds Ratio	95% CI	Beta	*P*-Value	Odds Ratio	95% CI	Beta
C4A	0.213	0.808	[0.574–1.125]	−0.212	0.285	0.838	[0.601–1.155]	−0.176
C4B	0.739	1.064	[0.736–1.540]	0.062	0.760	0.945	[0.656–1.352]	−0.056
C4L	0.543	0.927	[0.727–1.180]	−0.074	0.212	0.857	[0.669–1.088]	−0.153
C4S	0.908	0.982	[0.725–1.328]	−0.017	0.744	1.050	[0.781–1.417]	0.049
C4AL	0.840	0.964	[0.676–1.375]	−0.036	0.754	0.944	[0.661–1.346]	−0.056
C4AS	0.100	0.489	[0.180–1.003]	−0.715	0.150	0.640	[0.324–1.126]	−0.444
C4BL	0.775	0.953	[0.686–1.316]	−0.047	0.389	0.869	[0.629–1.193]	−0.140
C4BS	0.594	1.088	[0.795–1.491]	0.085	0.432	1.132	[0.831–1.551]	0.124
C4A expression	0.221	0.667	[0.345–1.267]	−0.403	0.158	0.629	[0.325–1.184]	−0.462
C4B expression	0.822	1.044	[0.710–1.533]	0.043	0.837	0.962	[0.663–1.387]	−0.038

2								
Females (*N* = 108)								
C4A	0.982	1.006	[0.560–1.812]	0.006	0.621	1.161	[0.639–2.124]	0.149
C4B	0.071	0.551	[0.276–1.029]	−0.596	0.156	0.629	[0.319–1.175]	−0.463
C4L	0.962	0.990	[0.674–1.453]	−0.009	0.901	0.975	[0.657–1.438]	−0.024
C4S	0.175	0.716	[0.436–1.153]	−0.333	0.570	0.870	[0.536–1.408]	−0.138
C4AL	0.858	1.056	[0.575–1.955]	0.055	0.617	1.171	[0.627–2.203]	0.158
C4AS	0.477	0.568	[0.106–2.723]	−0.564	0.825	0.837	[0.173–4.505]	−0.176
C4BL	0.835	0.948	[0.576–1.560]	−0.052	0.571	0.864	[0.518–1.432]	−0.145
C4BS	0.244	0.747	[0.452–1.214]	−0.291	0.610	0.881	[0.539–1.438]	−0.126
C4A expression	0.860	0.906	[0.302–2.697]	−0.097	0.922	1.056	[0.344–3.193]	0.054
C4B expression	0.113	0.583	[0.287–1.111]	−0.538	0.188	0.638	[0.315–1.224]	−0.448

2. Sex-stratified logistic regression analysis between C4 variants and suicide attempt/ideation in females with age and ancestry covariate.

**Fig. 5:**
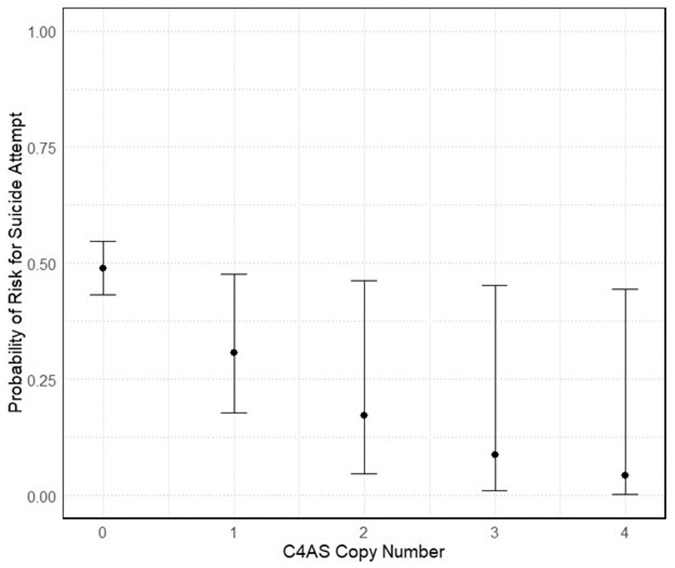
Probability of suicide attempt with increasing *C4*AS copy number. The risk of suicide attempts decreases from 49% (*C4*AS copy number = 0) to 5% (*C4*AS = 4). The inferential error bars widen with increasing *C4*AS copy numbers, indicating a higher uncertainty, due to a decreasing sample of cases with higher *C4*AS copy numbers. The adjusted probabilities were calculated based on our logistic regression model (*N* = 391) with age, sex, and ancestry as covariates. Supplementary Table 3 includes detailed measurements.

The presented outcome corresponds to the logistic regression model incorporating age, sex, and ancestry (Europeans vs non-Europeans) as covariates. The results between *C4*AS copy number and suicide attempt retained their marginal significance even after the inclusion of substance abuse and alcohol abuse as additional covariates in the model (Supplementary Table 1). In addition, the unadjusted analysis before controlling for covariates is also reported in Supplementary Table 2. Figure 5 depicts the probability of suicide attempts with increasing copy numbers of C4AS based on our logistic regression model.

### Sensitivity analysis

Through a detailed examination of the *C4*AS copy number distribution between suicide attempters and non-attempters (Fig. 3b), it becomes apparent that the occurrence of one or more *C4*AS copy numbers is infrequent. To mitigate the potential influence of outliers on the significance of the association between *C4*AS and suicide attempt/ideation, individuals with one or more copies of *C4*AS were combined into a pooled group. This approach ensures a more robust analysis and strengthens the reliability of our findings regarding the relationship between *C4*AS and suicide attempt/ideation. The results of the sensitivity analysis remained marginally significant for suicide attempts (OR = 0.481, Beta = −0.730, CI: [0.203–1.051], *p* = 0.077) but not for suicidal ideation (OR = 0.655, Beta = −0.422, CI: [0.315–1.352], *p* = 0.251). This difference is likely attributed to some participants attempting suicide without any prior history of suicidal ideation.

### Sex-stratified analysis

To assess the sex-specific effect of C4 variants on suicide attempt/ideation, we stratified our sample into males (*N* = 283) and females (*N* = 108). The copy number of *C4*B shows some weak evidence of negative association with suicide attempts for females, but not for males (OR for females: 0.551, Beta = −0.596, 95% CI: [0.276–1.029], *p* = 0.071, Table 2.2; the OR for males = 1.064, Beta= 0.062, CI: [0.736–1.540], *p* = 0.739, Table 2.1), but in general, there is little evidence of variations across sexes.

## Discussion

There have been a few previous studies that have mostly analyzed copy number variants based on single-nucleotide polymorphism array data in relation to suicidality among schizophrenia patients^26–28^. However, this study is novel in examining the relationship between *C4* copy number variants and suicidality in schizophrenia using a direct-genotyping approach. We found a possible negative association between *C4*AS and suicide risk. Moreover, sex-stratified analyses revealed that there is a weak negative association for *C4*B in females, but not males, but in general, there was no strong difference between sexes. However, it should be noted that our study was exploratory in nature and had a modest sample size. Therefore, we relied on an estimation framework to observe general trends in the data, characterized by the effect size and confidence intervals.

Multiple lines of evidence suggest that increased *C4* expression is associated with schizophrenia susceptibility, may be due to excessive synaptic pruning^14,26,29^. Mouse models with *C4* overexpression have demonstrated reduced synaptic density in the medial prefrontal cortex, accompanied by abnormalities in glutamatergic cells^20,29^. These findings align with investigations in human subjects with a propensity for suicidal behaviour, where structural abnormalities in the prefrontal cortex have been observed alongside dysregulation in the glutamatergic neurotransmission system^27,28^. In addition, a recent in vivo positron emission tomography (PET) study in patients with schizophrenia has revealed aberrant changes in the frontal and anterior cingulate cortices^30^. These brain regions are known to be intricately involved in emotion regulation and have been implicated in the manifestation of suicidal ideation and behaviour^30,31^. Together, these integrated results suggest a potential role for *C4* involvement in causing alterations in synaptic connectivity in the prefrontal cortex in schizophrenia patients which could distort decision-making processes and potentially contribute to an increased vulnerability to suicidal behavior.

Considering the previously established link between *C4*A expression and an increased risk of schizophrenia^14^, we expected to observe a higher incidence of suicide among people with higher *C4*A copy numbers. Surprisingly, our analyses did not reveal any correlation between *C4*AL/predicted *C4*A expression and suicide attempts or suicidal ideation. Interestingly, we observed a negative association between *C4*AS copy number and both suicide attempts and suicidal ideation, indicating that a higher *C4*AS copy number was associated with fewer suicidal events. These results point to a potential protective effect of increased *C4*AS copies against suicidal events.

One potential explanation for this result is the effect of the HERV insertion on *C4* gene expression^20^. Previous studies have proposed that the HERV insertion may function as an enhancer of gene expression^32,33^. Consequently, it is plausible that in the absence of a HERV insertion in the *C4*AS variant, there could be reduced *C4*A, potentially mitigating neuro-abnormalities and contributing, at least in part, to the observed decrease in suicidal events. Moreover, in line with this potential explanation, it has been documented that *C4*L copy number has the opposite effect to *C4*S copy numbers^19^. Therefore, given the reduced incidence of suicide attempts and suicidal ideation observed in individuals with higher copies of *C4*AS, it is conceivable that the presence of *C4*AS may be associated with lower copies of *C4*AL, which is a stronger risk factor for schizophrenia risk^14^.

It has been previously shown that *C4* alleles could have a sex-specific effect on disease pathophysiology, specifically with schizophrenia as well as autoimmune disorders, including systemic lupus erythematosus and Sjögren’s syndrome. In all of these three illnesses, *C4* alleles influence men more strongly than women. At a protein level, *C4* and its effector *C3*, have been shown to be present at higher levels in the cerebrospinal fluid and plasma of men compared to women, consistent with the more potent effects of *C4* in men, which could suggest a possible reason for men’s greater vulnerability to schizophrenia^34^. Our sex-stratified analyses revealed that increasing copy number of *C4*B is weakly associated with fewer suicide attempts and suicidal ideation in females but not males. This result may be explained by the potential protective effect of the *C4*B variant^34^, which may play a role in lowering suicidal events in females.

Our experimental approach to directly genotype the complex *C4* gene instead of computational methods provides a precise copy number for each compound structural variant. However, there were a number of limitations. Firstly, as the compound structural forms may have different configurations, we were unable to determine *C4* haplotypes. Also, due to the stringent DNA quality requirement of our experimental workflow, 21% of our samples failed to produce reliable genotyping data and were excluded from analyses. In addition, we could not determine the exact copy numbers for *C4*AL, *C4*BL, *C4*AS, or *C4*BS in about 1% of our genotyped samples. Secondly, our sample size was modest, raising concerns about potential type I and type II errors or the possibility that our lack of significance with *C4*A expression might be attributed to insufficient sample size. In addition, due to the retrospective nature of our study, we were unable to extensively characterize data on factors that may influence suicide risk (e.g., method for suicide attempt, intent, childhood trauma, and any changes in socioeconomic status). Additionally, the ancestry data used as a covariate in our analysis were self-reported, potentially introducing limitations in accounting for subtle variations within and between different populations. Therefore, further investigations with larger sample sizes are warranted to replicate and validate these findings. It should also be noted that schizophrenia is a highly polygenic disorder, and no single gene could explain a significant proportion of the disease risk by itself. Consequently, in order to conduct a more comprehensive analysis of all contributing factors to suicide risk in schizophrenia, it is important to consider additional genes related to suicide risk by utilizing methods such as polygenic risk scores. Overall, our preliminary findings provide encouraging evidence to warrant further exploration of the relationship between the *C4* gene and suicidal outcomes, with a specific focus on brain gene expression. Also, it is worth exploring the effect of other immune-related players in the complement pathway (e.g., CSMD1, C1q, C3) on suicide risk in schizophrenia patients.

## Methods

### Participants

A total of *N* = 433 subjects (mean age 38.7 ± 11.5; 71% males) with either schizophrenia or schizoaffective disorder were recruited as part of an ongoing schizophrenia genetic study at the Centre for Addiction and Mental Health (CAMH). Prior to study enrollment, all participants provided informed consent, and the research protocol was approved by the CAMH research ethics board. Eligible subjects were adults over the age of 18 who had a clinical diagnosis of either schizophrenia or schizoaffective disorder based on the Structured Clinical Interview for DSM-III-R or DSM-IV Axis (SCID)^35^. Exclusion criteria include head injury with loss of consciousness, seizure disorder, type II diabetes, and participants who were not able to read or understand English. The majority of participants were of self-reported European ancestry (sample characteristics detailed in Table 3). Data on lifetime suicide attempts, suicidal ideation, and suicidal plans were retrospectively extracted from a comprehensive search of the mood disorder module of the SCID, referral notes, life charts, family history, and a summary of medical records. The SCID specifically has a section (criterion A9 of major depressive episode) denoting suicidal ideation vs suicide plan (specific plan on how to commit suicide) vs suicide attempt. A suicide attempt was defined as any deliberate act of self-harm with intent for death^35^. This was specified as a suicide attempt in the hospital medical chart or recorded during the SCID interview by a well-trained clinician. Suicidal Ideation was determined based on Beck’s scale for suicidal ideation^36^. In addition, data on risk factors of suicide, such as substance use disorder and alcohol use disorder, were extracted. All participants provided saliva or blood samples for DNA extraction.

**Table 3:** Characteristics of participants used in the analysis

Characteristic	*n*
Mean age at assessment	38.7 ± 11.5
Sex (Male/Female)	311/122
Ancestry	
-European	303
-Non-European	130
Diagnosis	
-SCZ	371
-Schizoaffective disorder	63
Mean age of onset	21.4 ± 6.49
-Male (*n* = 267)	21.2 ± 6.3
-Female (*n* = 103)	22.0 ± 7.0
Suicide attempt count (*n* = 391)	173/391
-Male (*n* = 283)	113/283
-Female (*n* = 108)	60/108
Suicidal ideation count (*n* = 394)	236/394
-Male (*n* = 285)	168/285
-Female (*n* = 109)	68/109
*C4*A copy number	2.09 ± 0.742
*C4*B copy number	1.76 ± 0.677
*C4*L copy number	2.92 ± 1.054
*C4*S copy number	0.93 ± 0.856
*C4*AL copy number	1.98 ± 0.684
*C4*BL copy number	0.94 ± 0.789
*C4*AS copy number	0.11 ± 0.400
*C4*BS copy number	0.84 ± 0.820
*C4*A brain expression	1.17 ± 0.385
*C4*B brain expression	1.70 ± 0.656

Values are presented as mean ± SD.

### Genetic data collection

Genomic DNA was extracted from whole blood using the high-salt method^37^. To determine the precise copy number of each *C4* compound structural form (*C4*AL, *C4*BL, *C4*AS, and *C4*BS), we utilized a three-step approach similar to the Sekar et al. ^14^. paper. In step 1, Taqman-based copy number assays for the four structural elements [*C4*A (Hs07226349 _cn), *C4*B (Hs07226350_cn), *C4*L (Hs07226352_cn), and *C4*S (Hs07226351_cn)] were run on the Viia 7 real-time PCR system (Thermo Fisher Scientific) in quadruplicate with RNaseP reference assay following manufacturer’s protocol, and the copy numbers of *C4* structural elements (*C4*A, *C4*B, *C4*L, *C4*S) were resolved using the CopyCaller software (Thermo Fisher Scientific). In step 2, in individuals with at least one copy of *C4*S, standard long-range PCR was performed with primers specific to *C4*S: forward 5′-TCAGCATGTACAGACAGGAATACA-3′ and reverse 5′-GAGTGCCACAGTCTCATCATTG-3′ (TaKaRa, Clontech)^38^. In step 3, using a custom-designed Taqman genotyping assay, we determined the presence of *C4*A and/or *C4*B in the *C4*S long-range PCR product (Thermo Fisher Scientific). Subsequently, we determined the copy number of *C4*AS and *C4*BS which allowed us to extrapolate the copy numbers of *C4*AL and *C4*BL through subtraction of the total copies of *C4*A, *C4*B, *C4*L, and *C4*S. To assess genotyping quality, the formula [*C4*A + *C4*B = *C4*L + *C4*S] was used, and samples with unmatching numbers were re-genotyped. Samples with failed second genotyping were excluded from the analysis. Moreover, we calculated the predicted *C4*A and *C4*B brain expression using the formula provided in Sekar et al.’s paper^14^.

$$\begin{array}{c}C4A\,{expression}=(0.47* C4{AL})+(0.47* C4{AS})+(0.20* C4{BL})\\ C4B\,{expression}=(1.03* C4{BL})+(0.88* C4{BS})\end{array}$$

### Statistical analysis

Logistic regression models were used to examine the association between copy numbers of *C4* structural forms (*C4*A, *C4*B, *C4*L, *C4*S), compound structural forms (*C4*AL, *C4*BL, *C4*AS, *C4*BS), and the predicted brain expression level of C4A and C4B with suicide attempt and suicidal ideation. Factors affecting suicidality such as sex, age, substance abuse, and alcohol abuse were used as covariates. In addition, due to the previously established sex-specific association of *C4* to schizophrenia and other autoimmune disorders, sex-stratified analyses were conducted to assess the sex-specific effect of *C4* variants on suicidal outcomes. Data analyses and visualization were conducted using RStudio (version 2022.07.0 + 548). A power analysis conducted with the software GPower and using a logistic regression model, with two-sided tests, confidence level alpha 0.05 and prevalence of the outcomes encountered in our data showed that with our sample size, we have 80% to detect effect sizes equivalent to odds ratio 0.37, or a prevalence change from 44% to 22%. This is a large effect and would make statistical significance testing underpowered, particularly under multiple testing adjustments. For this reason, we will use an estimation framework for statistical inference, where we focus on the reporting of effect sizes and their precision (95% confidence intervals) and the interpretation of overall patterns of association instead of focusing on *p* < 0.05. We still report p-values descriptively, as a measure of statistical evidence.

### Supplementary information

Supplemental Material

## Data Availability

The dataset for this manuscript is not publicly available because of issues of data ownership and participant consent. The data used to support the findings of this study are available from the corresponding author upon reasonable request.

## Code availability

The codes used to support the findings of this study are available from the corresponding author upon reasonable request.
