# Supplementary material for: Association study of the complement component C4 gene and suicide risk in schizophrenia
Source: Schizophrenia (Heidelb). 2024 Feb 10;10(1):14. doi: 10.1038/s41537-024-00440-w (PMC10858919; doi:10.1038/s41537-024-00440-w)
Supplement: Supplementary file 1 — Supplemental Material [file 41537_2024_440_MOESM1_ESM.pdf]

Supplementary Material:

Table 1. Logistic regression analyses of *C4* variants and suicide attempt/ideation in males and females with age, sex, substance abuse, and alcohol abuse as covariates.

**Males and Females** (*N*=380)								
**C4 Variant**	**Suicide Attempt**				**Suicidal Ideation**			
	P-value	Odds Ratio	95% CI	Beta	P-Value	Odds Ratio	95% CI	Beta
C4A	0.439	0.892	[0.664-1.189]	-0.114	0.419	0.887	[0.661-1.183]	-0.119
C4B	0.496	0.896	[0.651-1.227]	-0.109	0.588	0.916	[0.666-1.253]	-0.086
C4L	0.996	0.999	[0.815-1.224]	-0.0004	0.299	0.896	[0.727-1.099]	-0.108
C4S	0.219	0.851	[0.657-1.099]	-0.160	0.882	1.019	[0.790-1.316]	0.019
C4AL	0.827	1.034	[0.759-1.411]	0.034	0.925	0.985	[0.718-1.347]	-0.014
C4AS	0.063	0.491	[0.213-0.954]	-0.711	0.136	0.645	[0.342-1.105]	-0.438
C4BL	0.965	1.005	[0.765-1.319]	0.005	0.388	0.886	[0.673-1.164]	-0.120
C4BS	0.588	0.929	[0.711-1.211]	-0.073	0.537	1.087	[0.834-1.420]	0.083
C4A Expression	0.452	0.808	[0.461-1.403]	-0.212	0.225	0.706	[0.397-1.231]	-0.347
C4B Expression	0.591	0.913	[0.655-1.268]	-0.090	0.697	0.938	[0.676-1.294]	-0.063

Table 2. Logistic regression analyses between *C4* variants and suicide attempt/ideation in males and females without any covariates included (unadjusted analysis)

**Males and Females** (*N*=391)								
**C4 Variant**	**Suicide Attempt**				**Suicidal Ideation**			
	P-value	Odds Ratio	95% CI	Beta	P-Value	Odds Ratio	95% CI	Beta
C4A	0.155	0.820	[0.621-1.074]	-0.198	0.260	0.855	[0.650-1.121]	-0.155
C4B	0.709	0.945	[0.703-1.268]	-0.055	0.333	0.863	[0.638-1.161]	-0.147
C4L	0.973	0.996	[0.821-1.209]	-0.003	0.594	0.948	[0.780-1.151]	-0.052
C4S	0.134	0.834	[0.657-1.055]	-0.180	0.269	0.875	[0.691-1.108]	-0.132
C4AL	0.828	0.967	[0.720-1.299]	-0.032	0.928	0.986	[0.732-1.326]	-0.013
C4AS	0.024*	0.437	[0.196-0.830]	-0.827	0.040*	0.557	[0.302-0.937]	-0.584
C4BL	0.709	1.049	[0.813-1.353]	0.048	0.834	0.973	[0.753-1.258]	-0.027
C4BS	0.555	0.928	[0.723-1.187]	-0.074	0.723	0.956	[0.747-1.225]	-0.044
C4A Expression	0.219	0.718	[0.421-1.212]	-0.330	0.207	0.711	[0.415-1.203]	-0.340
C4B Expression	0.854	0.971	[0.713-1.321]	-0.028	0.521	0.904	[0.663-1.228]	-0.100

Table 3. Probability of risk for suicide attempt and suicidal ideation with increasing *C4*AS copy number based on our statistical model.

***C4*AS Copy number**	**Probability of Risk for Suicide Attempt**	**Confidence Interval**	**Probability of Risk for Suicidal Ideation**	**Confidence Interval**
**0**	0.489	0.431-0.547	0.573	0.510-0.634
**1**	0.308	0.178-0.476	0.467	0.335-0.604
**2**	0.171	0.047-0.463	0.363	0.162-0.627
**3**	0.087	0.011-0.453	0.271	0.068-0.653
**4**	0.042	0.002-0.443	0.195	0.026-0.681
